# Changes in active commuting and changes in physical activity in adults: a cohort study

**DOI:** 10.1186/s12966-015-0323-0

**Published:** 2015-12-18

**Authors:** Louise Foley, Jenna Panter, Eva Heinen, Richard Prins, David Ogilvie

**Affiliations:** MRC Epidemiology Unit and UKCRC Centre for Diet and Activity Research (CEDAR), University of Cambridge School of Clinical Medicine, Box 285, Cambridge Biomedical Campus, Cambridge, CB2 0QQ UK

**Keywords:** Active travel, Active commuting, Walking, Bicycling, Physical activity, Self-report, Actiheart, Effect modifier

## Abstract

**Background:**

Active travel is associated with greater physical activity, but there is a dearth of research examining this relationship over time. We examined the longitudinal associations between change in time spent in active commuting and changes in recreational and total physical activity.

**Methods:**

Adult commuters working in Cambridge, United Kingdom completed questionnaires in 2009 and 2012, and a sub-set completed objective physical activity monitoring in 2010 and 2012. Commuting was assessed using a validated seven-day travel to work record. Moderate-to-vigorous physical activity was assessed using the Recent Physical Activity Questionnaire and combined heart rate and movement sensing. We used multivariable multinomial logistic regression models to examine associations between change in time spent in active commuting and tertiles of changes in time spent in recreational and total physical activity.

**Results:**

Four hundred sixty-nine participants (67 % female, mean age 44 years) provided valid travel and self-reported physical activity data. Seventy-one participants (54 % female, mean age 45 years) provided valid travel and objectively measured physical activity data. A decrease in active commuting was associated with a greater likelihood of a decrease in self-reported total physical activity (relative risk ratio [RRR] 2.1, 95 % CI 1.1, 4.1). Correspondingly, an increase in active commuting was associated with a borderline significantly greater likelihood of an increase in self-reported total physical activity (RRR 1.8, 95 % CI 1.0, 3.4). No associations were seen between change in time spent in active commuting and change in time spent in either self-reported recreational physical activity or objectively measured physical activity.

**Conclusions:**

Changes in active commuting were associated with commensurate changes in total self-reported physical activity and we found no compensatory changes in self-reported recreational physical activity. Promoting active commuting has potential as a public health strategy to increase physical activity. Future longitudinal research would be useful to verify these findings.

**Electronic supplementary material:**

The online version of this article (doi:10.1186/s12966-015-0323-0) contains supplementary material, which is available to authorized users.

## Background

Travel is a commonplace daily activity [1, 2]. In the United Kingdom (UK), 20 % of journeys are less than one mile in length [1]. Promoting active travel (walking or cycling to get from one place to another), which may include active commuting (walking or cycling to get to or from work), has considerable potential as a feasible and pragmatic public health strategy to enable people to accumulate daily physical activity. In adults, active travel is associated with greater self-reported and objectively measured physical activity [3–6], improved health and reduced mortality [3, 7, 8].

Increasing one type of physical activity such as active travel does not necessarily result in increased total physical activity if activity in one domain is substituted for activity in another. For example, if walking to work displaces a recreational morning walk, total physical activity—and the health implications of such—may remain unchanged. However, research conducted in the UK suggests that active travel tends to be undertaken in addition to, not instead of, recreational physical activity, and is associated with commensurate increases in total physical activity. In a cross-sectional study, total weekday physical activity measured by accelerometer was 60 % higher in those commuting on foot than in those commuting by car, with no differences between groups in weekend physical activity or sedentary behaviour [6]. Similarly, in another study, women who reported 150 min or more of weekly active commuting accumulated an additional 8.5 daily minutes of accelerometer-measured physical activity on average than those who reported no active commuting [9]. In a third cross-sectional study, there were no differences in self-reported recreational physical activity between those using active modes of transport and those using motorised modes only or a combination of modes, and total self-reported physical activity was 400 min per week higher on average in those reporting more than 150 min of active travel per week than in those reporting none [10]. Longitudinal analyses from the same study indicated that total self-reported physical activity increased by approximately 100 min per week on average in those whose active travel increased compared with those whose active travel remained unchanged, with no differences between these groups in time spent in recreational physical activity [11].

While initial findings are promising, only one previous study [11] has examined the longitudinal relationship between change in active travel and changes in self-reported recreational and total physical activity in adults. Self-report of physical activity has known limitations due to recall and social biases [12] but has the advantage of being able to identify specific activities undertaken across different domains of physical activity (e.g. transport, recreational and occupational) [13], which is important for assessing the possibility of behavioural substitution. Conversely, objective measurement of physical activity overcomes some of these limitations but is currently not able to reliably identify specific activities; for studies of active travel, it is also important that some devices (e.g. accelerometers and pedometers) have known limitations for assessing cycling [14]. The use of multiple approaches may therefore provide complementary information. In addition, the relationship between active travel and physical activity is unlikely to be consistent across all population groups. Investigating moderating effects may illuminate the nuances of this relationship, and in particular highlight groups that stand to benefit least or most from increasing active commuting.

Understanding if and how active travel and other physical activity behaviours change in tandem has important implications for the value of promoting, or creating policy to promote, active travel. Therefore, the primary aim of this study was to examine the longitudinal associations between change in active commuting and changes in recreational and total physical activity (both self-reported and objectively measured) in adults. The secondary aim was to explore potential moderators of these relationships.

## Methods

### Design

Data were collected in four annual surveys between 2009 (T1) and 2012 (T4), as part of the Commuting and Health in Cambridge cohort study of working adults in Cambridge, UK. Ethical approval for the study was obtained from the Hertfordshire Research Ethics Committee (reference numbers 08/H0311/208, 09/H0311/116 and 10/H0311/65) and the Cambridge Psychology Research Ethics Committee (reference number 2012.14). Each participant provided written informed consent.

The study methods [15], questionnaire and characteristics of the baseline sample [16] have been described in detail elsewhere and are summarised briefly below.

### Participants

Participants were aged 16 or over, lived within a radius of approximately 30 km of Cambridge city centre, and worked in Cambridge. Recruitment occurred primarily via workplaces, with a range of geographic workplace settings (e.g. city centre and city fringe) and workplace types (e.g. hospitals, universities, local authorities and retail outlets) included.

### Procedure

Each year, beginning at baseline (T1) in 2009, participants completed a postal questionnaire collecting information on travel behaviour, physical activity and health, as well as number of individual and socio-demographic factors. In addition, a subset of participants were invited to participate in seven days of objective physical activity monitoring, which at T2, T3 and T4 included wearing a combined heart rate and movement sensor. Each annual survey was conducted at the same time of year (May to October), and individual assessments were matched to the nearest week of the year if possible to minimise the influence of seasonal variation on travel and physical activity behaviour.

### Measurement

#### Self-reported commuting behaviour

Commuting behaviour was assessed using a seven-day retrospective travel to work record. Our instrument was based on one shown in previous research to have acceptable test-retest reliability [17], and was shown in the current study to have good agreement with objectively derived estimates of active commuting time [18]. For each of the seven days, participants were asked to report whether they travelled to work and the mode(s) of transport used for commuting in both directions. Participants could report both single- (e.g. car) and multi-modal (e.g. bus and walking) journeys. For walking and cycling only, participants reported the typical duration of these portions of the journey if they used either mode. From these estimates, weekly time spent in active commuting (walking plus cycling) was calculated, as well as in cycle and walking commuting separately.

#### Self-reported physical activity

Moderate-to-vigorous physical activity (referred to hereafter as physical activity) was assessed using the Recent Physical Activity Questionnaire (RPAQ). The RPAQ is a retrospective recall of physical activity undertaken in the previous four weeks across domestic, occupational, recreational and transport domains. Recreational and total physical activity were derived by summing the time spent in activities classified above three metabolic equivalents (METs) across the recreational or all domains respectively, using standard procedures [19]. The RPAQ is based on a previously validated questionnaire [20] and performs comparably to other self-report questionnaires in terms of reliability [19], criterion validity against estimates of energy expenditure using the doubly-labelled water method [19], and convergent validity against estimates of physical activity derived from combined heart rate and movement sensing [21].

#### Objectively measured physical activity

Physical activity was assessed using combined heart rate and movement sensors. Participants were asked to wear a combined sensor (Actiheart, CamNtech, Papworth, UK) for seven days [22]. Heart rate data were cleaned [23] and activity intensity was derived from a combination of movement registration and heart rate, using a branched equation framework [24]. Individual calibration of heart rate data was based on age, sex and sleeping heart rate [25], using a group equation derived from a large cohort (n ~ 10,000) [26] of Cambridgeshire adults. Time spent above three METs was calculated using the Oxford 2005 [27] equation for the estimation of resting metabolic rate.

#### Covariates

Participants reported their age, sex, number of cars or vans owned or available for use, access to a bicycle (yes/no) and distance from home to work (kilometres). Self-reported height and weight were used to compute body mass index (BMI). Baseline (T1) values of these variables, and baseline self-reported total physical activity from the RPAQ, were included as covariates in all models.

### Exposures and outcomes

For the analysis using self-reported physical activity data, the exposures were change between T1 and T4 in self-reported time spent in (a) active commuting (walking plus cycling; minutes/week), (b) cycle commuting (minutes/week) and (c) walking commuting (minutes/week). The outcomes were change between T1 and T4 in self-reported time spent in (i) total physical activity (minutes/week) and (ii) recreational physical activity (minutes/week). To maximise both the available sample size and the elapsed time period, we chose to use T1 and T4 data in this part of the analysis, though T2 and T4 data were used in the analysis of objective physical activity data described below due to the availability of combined heart rate and movement sensing data from T2 onwards.

For the analysis using objective physical activity data, the exposures were change between T2 and T4 in self-reported time spent in (a) active commuting (walking plus cycling; minutes/week), (b) cycle commuting (minutes/week) and (c) walking commuting (minutes/week). The outcome was change between T2 and T4 in objectively measured time spent in total physical activity (minutes/week).

Participants were included in each analysis if they provided valid data for both exposure and outcome at both time points. For self-reported commuting behaviour, data were considered invalid if the diary was blank and a “true zero”—such as annual leave or sickness absence—could not be verified (*n =* 29 for the T1-T4 analysis and *n =* 39 for the T2-T4 analysis), or if checking procedures raised concerns about data completeness (*n =* 3 for the T1-T4 analysis and *n =* 1 for the T2-T4 analysis). For self-reported physical activity (T1-T4 analysis), data were considered invalid where a participant did not provide a value for recreational or work physical activity (*n =* 5). Finally, for objectively measured physical activity (T2-T4 analysis), data were considered invalid if the participant did not wear the device for at least one day (*n =* 5).

Changes in exposures and outcomes were computed by subtracting the T1 or T2 value from the T4 value for each participant. Change in time spent in active commuting behaviour was not normally distributed because many participants reported no active commuting at one or both time points; therefore, change variables were categorised as “decreased” (delta < 0), “no change” (delta = 0) or “increased” (delta > 0) (see Additional file 1: Tables S1 and S2). Change in time spent in self-reported physical activity was also not normally distributed but could not be categorised in the same way because no participant had a delta value of zero; instead these variables were categorised into tertiles, with the highest category representing a “large increase” in physical activity (mean +390 [standard deviation (SD) 476] minutes/week for total physical activity and +323 [SD 455] minutes/week for recreational physical activity), and the lowest category representing a “large decrease” in physical activity (mean -495 [SD 596] minutes/week for total physical activity and -370 [SD 439] minutes/week for recreational physical activity). Change in time spent in objectively measured physical activity was similarly categorised into tertiles (“large increase” mean +283 [SD 239] minutes/week, “large decrease” mean -343 [SD 130] minutes/week). See Additional file 1: Tables S1 and S2.

### Analysis

Descriptive analysis of all variables was undertaken. T-tests, *χ*^2^ tests and signed-rank tests were used as appropriate to determine whether participants included in the analysis differed from those not included by a number of demographic characteristics. Signed-rank tests were used to evaluate whether exposures and outcomes had changed significantly over time.

Multivariable multinomial logistic regression analyses were carried out using Stata13 (Timberlake, London, UK) to assess the relationships between exposures and outcomes. Covariates were added progressively in steps, with the final model adjusted for age, sex, car ownership, access to a bicycle, distance from home to work, baseline BMI and baseline total physical activity. We tested all maximally adjusted models for the self-reported physical activity outcomes for interactions with age, sex, baseline BMI and baseline physical activity, which we hypothesised could act as moderators based on previous research [28]. We did not test for interactions in the analysis using objectively measured physical activity outcomes owing to the smaller sample size. For all analyses, the significance level was set at 0.05.

## Results

In total, 1164 participants returned a questionnaire at T1 (74 % of those invited to participate in the study); they had a mean age of 42 years (SD 13.5) and 68 % were female. Of these, 501 (43 %) also returned a questionnaire at T4. In addition, 201 participants wore combined heart rate and movement sensors at T2, of whom 79 (39 %) also wore devices at T4.

### Self-reported physical activity

Four hundred sixty-nine participants (67 % female; aged 20 to 71 years, mean age 44 [SD 11.1]) provided valid travel and physical activity data at both time points. Compared to those not included in this analysis, included participants were significantly older and had lower BMI, though there were no differences for sex, car ownership, bicycle access, distance from home to work or baseline physical activity. Small decreases over time in weekly active commuting, cycle commuting and walking commuting were found, as well as an overall decrease in total and recreational physical activity (Table 1). For active commuting (*p =* 0.001) and cycle commuting (*p =* 0.01), these reductions were statistically significant.

**Table 1:** Baseline demographics, active commuting and physical activity

Variable	*n*	Mean/*n*	SD/%
**Self-reported physical activity analysis**			
*Eligible*	*469*		
Sex	469		
Female		312	66.5
Male		157	33.5
Age	468	44.4	11.1
Number of cars owned	469	1.4	0.8
Access to a bicycle	466		
Yes		404	86.7
No		62	13.3
Distance from home to work (km)	468	12.6	11.0
Body mass index (kg/m^2^)	461	24.1	3.6
T1 active commuting (min/week)	463	127.5	116.8
T4 active commuting (min/week)	459	118.5	127.2
T1 cycle commuting (min/week)	468	93.0	115.7
T4 cycle commuting (min/week)	468	86.3	124.8
T1 walking commuting (min/week)	464	34.7	75.1
T4 walking commuting (min/week)	460	31.8	71.3
T1 self-reported total physical activity (min/week)	469	584.5	627.3
T4 self-reported total physical activity (min/week)	469	544.0	548.1
T1 self-reported recreational physical activity (min/week)	469	396.3	412.8
T4 self-reported recreational physical activity (min/week)	469	378.1	406.9
**Objectively measured physical activity analysis**			
*Eligible*	*71*		
Sex	71		
Female		38	53.5
Male		33	46.5
Age	71	44.6	10.1
Number of cars owned	71	1.5	0.8
Access to a bicycle	71		
Yes		63	88.7
No		8	11.3
Distance from home to work (km)	71	16.6	11.0
Body mass index (kg/m^2^)	69	24.5	3.3
T2 active commuting (min/week)	70	136.9	122.0
T4 active commuting (min/week)	67	128.2	131.0
T2 cycle commuting (min/week)	71	96.6	118.6
T4 cycle commuting (min/week)	71	85.7	124.6
T2 walking commuting (min/week)	70	40.6	87.1
T4 walking commuting (min/week)	67	41.5	84.6
T2 objectively measured total physical activity (min/week)	71	1020.8	472.1
T4 objectively measured total physical activity (min/week)	71	971.1	474.1

*%* percentage, *kg* kilograms, *km* kilometres, *m* metres, *min* minutes, *n* number, *SD* standard deviation

Time spent in active commuting increased in 30 % (*n =* 136), was maintained in 26 % (*n =* 120) and decreased in 43 % (*n =* 197) of participants. Results of the multivariable multinomial logistic regression models are displayed in Table 2. In the maximally adjusted model, a decrease in active commuting was associated with a greater likelihood of a large decrease in total physical activity (relative risk ratio [RRR] 2.1, 95 % confidence interval [CI] 1.1, 4.1). Correspondingly, an increase in active commuting was associated with a borderline significantly greater likelihood of a large increase in total physical activity (RRR 1.8, 95 % CI 1.0, 3.4). Time spent in cycle commuting increased in 23 % (*n =* 108), was maintained in 45 % (*n =* 212) and decreased in 31 % (*n =* 147) of participants. In the maximally adjusted model, an increase in cycle commuting was associated with a greater likelihood of both a large increase (RRR 2.5 95 % CI 1.2, 5.0) and a large decrease (RRR 3.0, 95 % CI 1.4, 6.3) in total physical activity. Finally, time spent in walking commuting increased in 17 % (*n =* 76), was maintained in 65 % (*n =* 296) and decreased in 18 % (*n =* 83) of participants. No significant associations were seen between changes in walking commuting and total physical activity, or between any of the active commuting exposures and recreational physical activity in the maximally adjusted models, though there was some indication of an association between an increase in cycle commuting and a large decrease in recreational physical activity.

**Table 2:** Associations between change in active commuting and changes in self-reported physical activity between T1 and T4

	RRR (95 % CI)			
Exposure: Change in active commuting (min/week; no change, increase, decrease)	Model 1	Model 2	Model 3	Model 4
Outcome: Change in self-reported physical activity (min/week; tertiles)				
**Total physical activity**				
*Total physical activity mid tertile (~no change)*	*Ref*	-	-	-
Total physical activity top tertile (~increase)				
Active commuting				
Increase	1.8 (1.0, 3.2)*	1.7 (0.9, 3.1)	1.7 (0.9, 3.2)	1.8 (1.0, 3.4)****
Decrease	1.4 (0.8, 2.5)	1.4 (0.8, 2.5)	1.4 (0.8, 2.5)	1.4 (0.8, 2.6)
Total physical activity bottom tertile (~decrease)				
Active commuting				
Increase	2.2 (1.2, 4.1)*	2.1 (1.1, 4.0)*	2.1 (1.1, 4.1)*	1.8 (0.9, 3.7)
Decrease	2.8 (1.6, 5.0)***	2.8 (1.6, 5.1)***	2.6 (1.4, 4.8)**	2.1 (1.1, 4.1)*
*Total physical activity mid tertile (~no change)*	*Ref*	-	-	-
Total physical activity top tertile (~increase)				
Cycle commuting				
Increase	2.6 (1.4, 4.7)**	2.4 (1.3, 4.4)**	2.5 (1.3, 4.9)**	2.5 (1.2, 5.0)*
Decrease	1.1 (0.6, 1.8)	1.0 (0.6, 1.7)	1.1 (0.6, 1.9)	1.0 (0.6, 1.9)
Total physical activity bottom tertile (~decrease)				
Cycle commuting				
Increase	4.1 (2.2, 7.7)***	3.8 (2.0, 7.2)***	3.9 (2.0, 7.7)***	3.0 (1.4, 6.3)**
Decrease	2.4 (1.4, 4.0)**	2.3 (1.3, 3.8)**	2.1 (1.2, 3.8)*	1.5 (0.8, 2.9)
*Total physical activity mid tertile (~no change)*	*Ref*	-	-	-
Total physical activity top tertile (~increase)				
Walking commuting				
Increase	1.0 (0.5, 1.8)	0.9 (0.5, 1.7)	0.9 (0.5, 1.7)	1.0 (0.5, 1.9)
Decrease	1.3 (0.7, 2.5)	1.3 (0.7, 2.4)	1.3 (0.7, 2.5)	1.4 (0.7, 2.7)
Total physical activity bottom tertile (~decrease)				
Walking commuting				
Increase	0.8 (0.4, 1.5)	0.8 (0.4, 1.5)	0.9 (0.4, 1.6)	0.9 (0.5, 1.9)
Decrease	1.4 (0.8, 2.5)	1.4 (0.8, 2.6)	1.5 (0.8, 2.8)	1.8 (0.9, 3.6)
**Recreational physical activity**				
*Recreational physical activity mid tertile (~no change)*	*Ref*	-	-	-
Recreational physical activity top tertile (~increase)				
Active commuting				
Increase	1.0 (0.5, 1.7)	0.9 (0.5, 1.7)	1.0 (0.5, 1.9)	1.0 (0.5, 1.9)
Decrease	1.0 (0.6, 1.8)	1.0 (0.6, 1.8)	1.0 (0.6, 1.8)	1.0 (0.6, 1.8)
Recreational physical activity bottom tertile (~decrease)				
Active commuting				
Increase	1.6 (0.9, 3.0)	1.6 (0.9, 3.0)	1.7 (0.9, 3.3)	1.6 (0.8, 3.0)
Decrease	1.3 (0.7, 2.3)	1.3 (0.7, 2.3)	1.3 (0.7, 2.4)	1.0 (0.5, 1.9)
*Recreational physical activity mid tertile (~no change)*	*Ref*	-	-	-
Recreational physical activity top tertile (~increase)				
Cycle commuting				
Increase	1.1 (0.6, 2.1)	1.1 (0.6, 2.0)	1.1 (0.6, 2.2)	1.0 (0.5, 2.0)
Decrease	0.9 (0.6, 1.5)	0.9 (0.5, 1.5)	0.9 (0.5, 1.6)	0.8 (0.4, 1.4)
Recreational physical activity bottom tertile (~decrease)				
Cycle commuting				
Increase	2.1 (1.2, 3.7)*	2.0 (1.1, 3.6)*	2.0 (1.1, 3.8)*	1.5 (0.8, 3.0)
Decrease	1.1 (0.7, 1.8)	1.1 (0.6, 1.8)	1.0 (0.5, 1.8)	0.6 (0.3, 1.2)
*Recreational physical activity mid tertile (~no change)*	*Ref*	-	-	-
Recreational physical activity top tertile (~increase)				
Walking commuting				
Increase	0.8 (0.4, 1.4)	0.7 (0.4, 1.4)	0.8 (0.4, 1.4)	0.8 (0.4, 1.5)
Decrease	1.0 (0.5, 1.9)	1.0 (0.5, 1.8)	1.0 (0.5, 1.8)	1.0 (0.6, 1.9)
Recreational physical activity bottom tertile (~decrease)				
Walking commuting				
Increase	0.9 (0.5, 1.7)	0.9 (0.5, 1.7)	1.0 (0.5, 1.8)	1.1 (0.6, 2.1)
Decrease	1.3 (0.7, 2.3)	1.3 (0.7, 2.4)	1.3 (0.7, 2.4)	1.5 (0.8, 2.9)

Model 1 is unadjusted

Model 2 is adjusted for age and sex

Model 3 is adjusted for variables in model 2 plus car ownership, access to a bicycle and self-reported distance between home and work

Model 4 is adjusted for variables in model 3 plus baseline body mass index and baseline self-reported total physical activity

*CI* confidence interval, *RRR* relative risk ratio

*p < 0.05, **p < 0.01, ***p < 0.001, ****p = 0.059

**Fig. 1:**
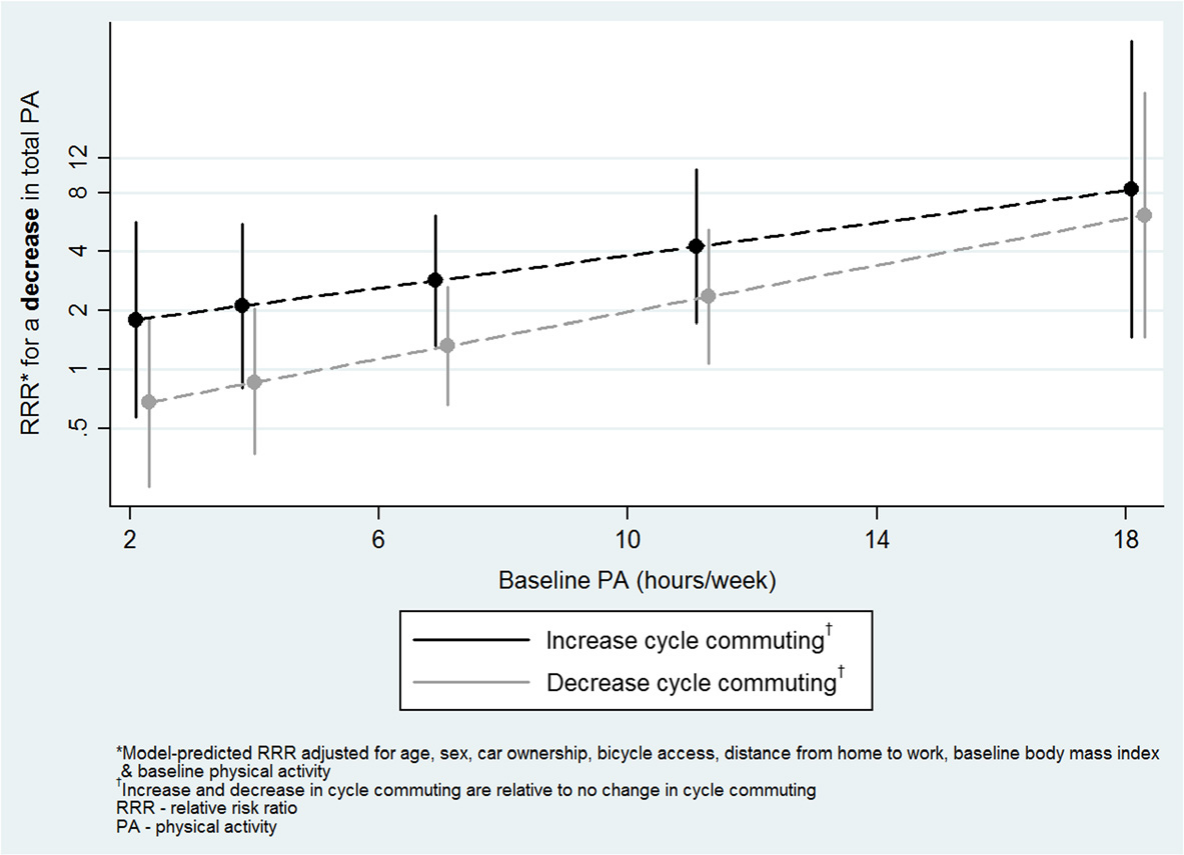
Significant moderation of the relationship between a decrease in cycle commuting and a decrease in self-reported total physical activity by baseline physical activity

**Fig. 2:**
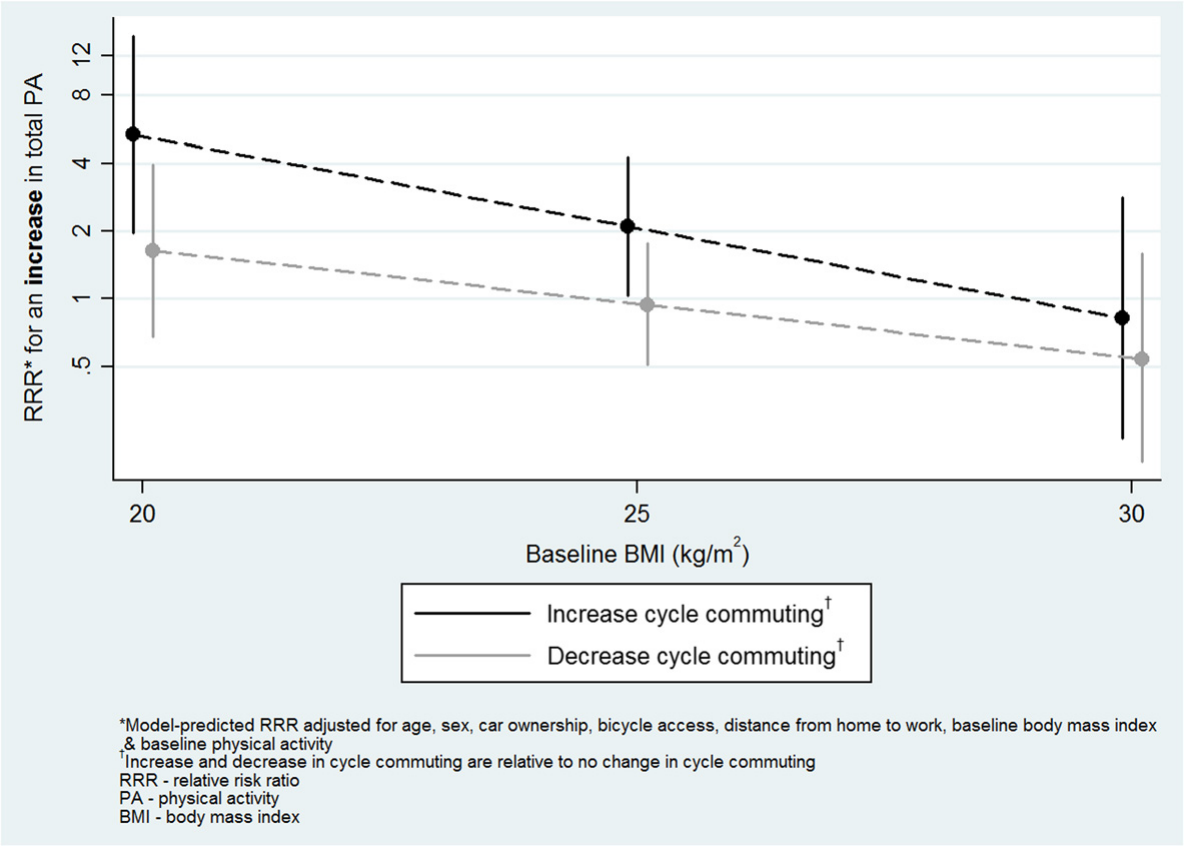
Significant moderation of the relationship between an increase in cycle commuting and an increase in self-reported total physical activity by baseline body mass index

**Fig. 3:**
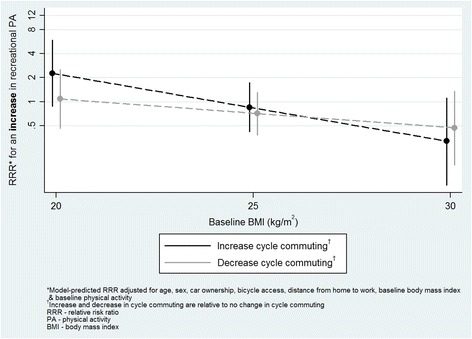
Significant moderation of the relationship between an increase in cycle commuting and an increase in self-reported recreational physical activity by baseline body mass index

**Fig. 4:**
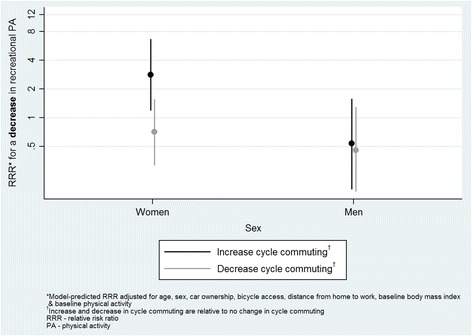
Significant moderation of the relationship between an increase in cycle commuting and a decrease in self-reported recreational physical activity by sex

For cycle commuting, significant interactions were found with baseline physical activity, BMI and sex. At higher levels of baseline physical activity, decreasing cycle commuting (relative to no change) was more strongly related to a decrease in total physical activity (RRR 1.1, 95 % CI 1.0, 1.3; Fig. 1). In addition, a post-hoc *t*-test indicated that participants whose cycle commuting increased and whose total physical activity decreased reported significantly higher baseline physical activity than those for whom both increased (930 vs. 471 min/week respectively, *p =* 0.001). An increase in cycle commuting was less likely to be related to an increase in total or recreational physical activity at higher baseline BMI (RRR 0.8, 95 % CI 0.7, 1.0 for both total and recreational physical activity; Figs. 2 and 3). Finally, an increase in cycle commuting was more strongly related to a decrease in recreational physical activity in women than men (RRR 5.3, 95 % CI 1.4, 19.8; Fig. 4).

For active commuting and walking commuting, an inconsistent pattern of interactions emerged, which is summarised in Additional file 2.

### Objectively measured physical activity

Seventy-one participants (54 % female; aged 23 to 64 years, mean age 45 [SD 10.1]) provided valid travel and objectively measured physical activity data at both time points. Compared to those not included in this analysis, included participants were significantly older, more likely to be male, and had a longer distance from home to work, though there were no differences for car ownership, bicycle access, baseline BMI or baseline physical activity. Small decreases over time in weekly active commuting, cycle commuting and objectively measured physical activity were evident, as well as a small increase in walking commuting (Table 1). None of these changes were statistically significant.

In all models, no significant associations were seen between changes in any of the active commuting exposures and time spent in objectively measured physical activity (Table 3).

**Table 3:** Associations between change in active commuting and changes in objectively measured physical activity between T2 and T4

	RRR (95 % CI)			
Exposure: Change in active commuting (min/week; no change, increase, decrease)	Model 1	Model 2	Model 3	Model 4
Outcome: Change in objectively measured physical activity (min/week; tertiles)				
**Total physical activity**				
*Total physical activity mid tertile (~no change)*	*Ref*	-	-	-
Total physical activity top tertile (~increase)				
Active commuting				
Increase	1.1 (0.2, 6.4)	1.2 (0.2, 7.5)	1.1 (0.2, 7.5)	1.4 (0.2, 11.0)
Decrease	1.1 (0.2, 5.6)	1.1 (0.2, 5.7)	1.0 (0.2, 6.8)	1.1 (0.2, 7.3)
Total physical activity bottom tertile (~decrease)				
Active commuting				
Increase	0.7 (0.1, 3.8)	0.9 (0.1, 6.2)	0.5 (0.1, 4.2)	0.4 (0.0, 3.5)
Decrease	0.9 (0.2, 4.2)	0.9 (0.2, 4.8)	0.5 (0.1, 3.1)	0.4 (0.0, 2.7)
*Total physical activity mid tertile (~no change)*	*Ref*	-	-	-
Total physical activity top tertile (~increase)				
Cycle commuting				
Increase	1.9 (0.4, 9.0)	1.7 (0.3, 9.1)	2.4 (0.4, 14.7)	2.1 (0.3, 13.7)
Decrease	1.1 (0.3, 4.1)	1.1 (0.3, 4.0)	1.5 (0.3, 6.8)	1.3 (0.3, 5.9)
Total physical activity bottom tertile (~decrease)				
Cycle commuting				
Increase	2.1 (0.4, 10.5)	1.9 (0.4, 10.8)	1.7 (0.3, 10.8)	1.1 (0.2, 7.9)
Decrease	1.6 (0.4, 5.7)	1.4 (0.4, 5.3)	1.2 (0.3, 5.4)	0.9 (0.2, 4.2)
*Total physical activity mid tertile (~no change)*	*Ref*	-	-	-
Total physical activity top tertile (~increase)				
Walking commuting				
Increase	0.9 (0.2, 3.9)	0.9 (0.2, 4.4)	0.9 (0.2, 4.5)	1.2 (0.2, 7.0)
Decrease	3.2 (0.6, 19.0)	3.2 (0.5, 19.0)	3.0 (0.5, 18.6)	3.1 (0.5, 20.6)
Total physical activity bottom tertile (~decrease)				
Walking commuting				
Increase	0.9 (0.2, 3.9)	1.1 (0.3, 5.2)	1.2 (0.2, 5.9)	1.2 (0.2, 7.0)
Decrease	0.9 (0.1, 7.6)	0.8 (0.1, 7.1)	0.7 (0.1, 7.0)	0.6 (0.0, 6.5)

*CI* confidence interval, *RRR* relative risk ratio

Model 1 is unadjusted

Model 2 is adjusted for age and sex

Model 3 is adjusted for variables in model 2 plus car ownership, access to a bicycle and self-reported distance between home and work

Model 4 is adjusted for variables in model 3 plus baseline body mass index and baseline self-reported total physical activity

## Discussion

### Main findings

Our primary aim was to examine the longitudinal association between change in active commuting and change in physical activity. We found that changes in active commuting time were associated with commensurate changes in total self-reported physical activity. In addition, we found no compensatory change in self-reported recreational physical activity. This finding builds on previous cross-sectional studies [9, 10, 29] reporting positive associations between active travel and physical activity, and a single longitudinal study [11] which found comparable changes in active travel and physical activity in adults. Our study suggests that promoting active commuting has potential as a public health strategy to increase total physical activity.

When active modes of commuting were investigated separately, an increase in cycle commuting time was associated with both an increase and a decrease in total physical activity, with some evidence to suggest displacement of recreational physical activity. The divergent findings on total physical activity may be explained by distinct patterns of behaviour in different groups of participants. Testing of moderation (our secondary aim) indicated that those with higher baseline physical activity were less likely to “top up” decreases in cycle commuting with other physical activities. This finding may also suggest regression to the mean in highly active individuals, or be related to standard RPAQ cleaning procedures which involve truncating very high values. Women and individuals with higher BMI appeared more likely to displace physical activity from other domains to accommodate increases in cycle commuting. This is consistent with some (but not all) previous research [30], and could be taken to suggest that highly active or larger individuals, and women, may stand to benefit less from increasing cycle commuting. However—and importantly—the association between increased cycle commuting and increased total physical activity appeared to be more pronounced in those who were less active. From a public health perspective, improving physical activity in a low-active group is likely to result in greater health gain than encouraging those who already perform regular physical activity to do more.

Though the availability of combined heart rate and movement sensing data in this type of study is rare, the small sample size limited what we could learn from the analysis using objectively measured physical activity. The lack of significant associations may be related to a lack of statistical power, or may reflect a true lack of association between exposure and this outcome. Longitudinal examination of this relationship using objectively measured physical activity in larger samples is therefore an important avenue for future research. The descriptive pattern of a decrease in physical activity over time in the self-reported data was mirrored in the objective data. However, the numerical estimates of the volume of activity derived from combined heart rate and movement sensing were much higher. This probably reflects differences in what the tools actually measure (specific activities in the RPAQ, versus a physiological signal using combined heart rate and movement sensing) as well as the different time aggregation between methods: RPAQ involves the retrospective recall of a four-week period of physical activity in a single instance, whereas combined heart rate and movement sensing prospectively samples almost continuously and is thus able to capture tiny fluctuations in energy expenditure.

### Strengths and limitations

We tracked individual behaviour change over time in a large cohort of commuting adults. The strengths of the study include the relatively large sample size and the use of disaggregated and total measures of commuting behaviour and physical activity. In addition, we explored the relationship between active commuting and physical activity using both self-reported and objective assessment of physical activity. The limitations of the study include the relatively low retention rate of the study cohort over time (though the rate was comparable to that of other similar studies [31, 32]), the potential for social desirability bias in the self-reported data, and the small sample size for the objective physical activity analysis. The sample was not representative of the UK population, with an over-representation of women and graduates, and Cambridge has a markedly higher prevalence of cycle commuting than the rest of the UK [33]. While the analyses suggest that a change in active commuting is associated with a change in self-reported physical activity, the direction of causality cannot be elucidated using this approach. Finally, the investigation into moderation indicated possible explanations for patterns exhibited in the data, but does not provide definitive answers.

### Implications

The findings have several implications for research and practice. The associations between active commuting and total physical activity appeared to be driven mainly by changes in cycle commuting. Though walking and cycling are often combined into an aggregate measure of active travel, the two modes had a different relationship with total physical activity in this study. In addition, the pattern of moderation was consistent for cycle commuting, but contradictory and not easily interpretable for walking commuting. These divergent findings may be related to aspects of the behaviours and their measurement. For example, walking commuting was much more stable than cycle commuting in this cohort. Two-thirds of participants maintained their weekly duration of walking commuting over time (the majority reported no walking at both time points), compared with less than half maintaining their weekly duration of cycle commuting. This resulted in smaller numbers of participants in the change categories (increase or decrease) in analyses using walking commuting as the exposure. Furthermore, walking is often an incidental component of a multi-modal journey, which may make walking more difficult to recall and report accurately [34]. Overall, the analyses reported here highlight the value of considering walking and cycling separately as well as in combination.

The relationship between cycle commuting and physical activity was divergent across different groups of participants. In particular, those who are not already highly active may be more likely to benefit from taking up cycle commuting from a physical activity perspective. These analyses suggest that, like many different strategies for promoting physical activity, encouraging active commuting may be successful in increasing overall physical activity in some but not all individuals, and that the potential population health gain cannot be inferred from a simple aggregate association.

## Conclusions

Changes in active commuting were associated with commensurate changes in total self-reported physical activity and we found no compensatory change in self-reported recreational physical activity. Promoting active commuting has potential as a public health strategy to increase physical activity. Future longitudinal research would be useful to verify these findings.

## Additional files

Additional file 1:
**Change categories for active commuting and physical activity variables.** (DOC 33 kb) Additional file 2:
**Interactions for active commuting and walking commuting.** (DOC 22 kb)

## Abbreviations

%: Percentage
BMI: Body mass index
CI: Confidence interval
kg: Kilograms
km: Kilometres
m: Metres
METs: Metabolic equivalents
min: Minutes
n: Number
RPAQ: Recent Physical Activity Questionnaire
RRR: Relative risk ratio
SD: Standard deviation
UK: United Kingdom
