# Supplementary material for: Changes in active commuting and changes in physical activity in adults: a cohort study
Source: Int J Behav Nutr Phys Act. 2015 Dec 18;12:161. doi: 10.1186/s12966-015-0323-0 (PMC4683976; doi:10.1186/s12966-015-0323-0)
Supplement: Additional file 1: — Change categories for active commuting and physical activity variables. (DOC 33 kb) [file 12966_2015_323_MOESM1_ESM.doc]

Additional File 1

Table S1: Change categories for active commuting and physical activity variables between T1 and T4

| **Activity measure (min/week)** | **Mean (sd) change** | | |
| --- | --- | --- | --- |
|  | *Decrease* | *No change* | *Increase* |
| Self-reported active commuting | -84 (77) | 0 (0) | 84 (84) |
| Self-reported cycle commuting | -88 (78) | 0 (0) | 91 (90) |
| Self-reported walking commuting | -87 (77) | 0 (0) | 77 (79) |
|  | *Low tertile* | *Mid tertile* | *Top tertile* |
| Self-reported total physical activity | -495 (596) | -15 (51) | 390 (476) |
| Self-reported recreational physical activity | -370 (439) | -5 (41) | 323 (455) |

Table S2: Change categories for active commuting and physical activity variables between T2 and T4

| **Activity measure (min/week)** | **Mean (sd) change** | | |
| --- | --- | --- | --- |
|  | *Decrease* | *No change* | *Increase* |
| Self-reported active commuting | -74 (76) | 0 (0) | 101 (89) |
| Self-reported cycle commuting | -84 (69) | 0 (0) | 109 (95) |
| Self-reported walking commuting | -77 (99) | 0 (0) | 63 (64) |
|  | *Low tertile* | *Mid tertile* | *Top tertile* |
| Objective total physical activity | -343 (130) | -75 (82) | 283 (239) |
