# Supplementary material for: Changes in active commuting and changes in physical activity in adults: a cohort study
Source: Int J Behav Nutr Phys Act. 2015 Dec 18;12:161. doi: 10.1186/s12966-015-0323-0 (PMC4683976; doi:10.1186/s12966-015-0323-0)
Supplement: Additional file 2: — Interactions for active commuting and walking commuting. (DOC 22 kb) [file 12966_2015_323_MOESM2_ESM.doc]

Additional file 2

For active commuting, an interaction was found with baseline physical activity. At lower levels of baseline physical activity, a decrease in active commuting was more strongly related to a decrease in recreational physical activity (RRR 0.8, 95% CI 0.7, 1.0) – but also more strongly related to an increase in recreational physical activity relative to no change in active commuting (RRR 0.9, 95% CI 0.7, 1.0).

For walking commuting, interactions were found with baseline physical activity and age. Investigation of the significant interaction with baseline physical activity indicated that at lower levels of baseline physical activity, decreasing walking commuting was more strongly related to a decrease in total or recreational physical activity (RRR 0.8, 95% CI 0.7, 0.9 for total and RRR 0.9, 95% CI 0.8, 1.0 for recreational physical activity) – but also more strongly related to an increase in total physical activity (RRR 0.8, 95% CI 0.7, 1.0), relative to no change in walking commuting. At higher levels of baseline physical activity, decreasing walking commuting was less strongly related to an increase in recreational physical activity (RRR 0.8, 95% CI 0.7, 0.9). Finally, in older participants, a decrease in walking commuting was more strongly related to a decrease in recreational physical activity (RRR 1.1, 95% CI 1.0, 1.1).
